# Is diaphragm ultrasound better than rapid shallow breathing index for predicting weaning in critically ill elderly patients?

**DOI:** 10.5578/tt.20239701

**Published:** 2023-09-22

**Authors:** B. Er, B. Mızrak, A. Aydemir, S. Binay, C. Doğu, D. Kazancı, S. Turan

**Affiliations:** 1 Intensive Care Unit, University of Health Sciences Ankara Bilkent City Hospital, Ankara, Türkiye; 2 Department of Anesthesiology and Reanimation, University of Health Sciences Ankara Bilkent City Hospital, Ankara, Türkiye

**Keywords:** Extubation failure, diaphragm, ultrasonography

## Abstract

**ABSTRACT:**

Is diaphragm ultrasound better than rapid shallow breathing index for predicting weaning in critically ill elderly patients?

**Introduction:**

Prolonged weaning is associated with worse clinical outcomes in elderly patients. Beside traditional rapid shallow breathing index (RSBI), diaphragm ultrasound is a promising technique to evaluate the weaning process. We aimed to perform diaphragm ultrasonography for predicting the weaning process and its relation with frailty in the critically ill elderly population.

**Materials and Methods:**

We enrolled thirthy-two patients over 65 years of age who were mechanically ventilated for at least 48 hours. Thickness of diaphragm and excursion were evaluated within 48 h of intubation and during spontaneous breathing trial (SBT). Clinical parameters, frailty, diaphragm ultrasound results were compared according to the weaning status.

**Results:**

Mean age (standard deviation) was 79.3 ± 7.9 years, and 18 (56.3%) patients were classified as weaning failure. Diaphragmatic excursion during SBT was the only statistically significant parameter associated with weaning failure [2.37 cm (0.67) vs 1.43 cm (0.15), p= 0.0359]. There was no statistically s ignificant difference regarding RSBI between the groups [70.5 (46) vs 127.5 (80), p= 0.09]. Baseline thickness of diaphragm and excursion at SBT were moderately correlated with frailty.

**Conclusion:**

Ultrasound can be used to show diaphragm dysfunction in the elderly frail population, and a multifactorial approach to the extubation process may include ultrasound instead of using traditional RSBI alone.

## Abstract (tr)

**ÖZ:**

Yoğun bakımdaki yaşlı kırılgan hastalarda diyafram ultrasonu mekanik ventilatörden ayrılmayı hızlı yüzeyel solunum indeksine göre daha iyi öngördürebilir mi?

**Giriş:**

Mekanik ventilatörden ayrılma sürecindeki uzama, yaşlı hastalarda kötü klinik sonuçlarla ilişkili bir durumdur. Bu süreci değerlendirmek için kullanılan hızlı yüzeyel solunum indeksinin yanı sıra diyafram ultrasonografisi uygulaması umut verici bir tekniktir. Bu çalışmada yoğun bakımdaki ileri yaş hasta popülasyonunda diyafram kası ultrason ölçümleriyle birlikte ventilatörden ayrılma sürecini ve bunun kırılganlıkla ilişkisini araştırmayı amaçlandı.

**Materyal ve Metod:**

En az 48 saat mekanik ventilasyon uygulanan 65 yaş üstü 32 hastayı çalışmaya dahil edildi. Entübasyondan sonraki 48 saat içinde ve spontan solunum denemesi (SSD) sırasında diyaframın kalınlığı ve ekskürsiyonu değerlendirildi. Klinik parametreler, kırılganlık, diyafram ultrason sonuçları ventilatörden ayrılma durumuna göre karşılaştırıldı.

**Bulgular:**

Ortalama yaş (standart sapma) 79,3 ± 7,9 idi ve 18 (%56,3) hastada ventilatörden ayrılma süreci başarısız olarak değerlendirildi. SSD sırasında bakılan diyafragmatik ekskürsiyon, ayırma başarısızlığı ile ilişkili istatistiksel olarak anlamlı tek parametreydi [2,37 cm (0,67) - 1,43 cm (0,15), p= 0,035]. Başarılı ve başarısız gruplar arasında hızlı yüzeyel solunum indeksi açısından istatistiksel olarak anlamlı fark yoktu [70,5 (46)’e karşı 127,5 (80), p= 0,09]. Diyaframın kalınlığı ve SSD sırasındaki diyafram kalınlık ve ekskürsiyon değerleri kırılganlıkla orta derecede ilişkiliydi.

**Sonuç:**

Ultrason, yaşlı, kırılgan popülasyonda diyafram disfonksiyonunu göstermek için kullanılabilir ve ventilatörden ayrılma sürecini çok yönlü değerlendirmede diyafram ultrasonografisi katkı sağlayabilir.

## Introduction

The influence of the aging population on intensive care unit (ICU) admissions has expanded over the past few decades ( 1 ). This population is extremely difficult and has a high mortality rate due to their advanced age, the presence of frequent geriatric symptoms such frailty, cognitive decline, limited daily activity, and multiple concomitant illnesses. Frailty is associated with higher hospital and long term mortality in the ICU population ( 2 ). Loss of muscular mass, quality, and strength, known as the syndrome of sarcopenia, is more prevalent in older persons and is sometimes referred to as the physical manifestation of frailty ( 3 ).

Weaning failure has a complex pathophysiology that frequently involves concomitant conditions that affect the heart, lung, and respiratory muscles as well as the diaphragm, and it is associated with prolonged ICU stay and higher mortality ( 4 , 5 ). The diaphragm is the most significant respiratory muscle, and failure of this muscle is associated with a poor prognosis at the moment of liberation from mechanical ventilation ( 6 ). Bedside ultrasonography is a validated technique for detecting diaphragm dysfunction ( 7 ). Although rapid shallow breathing index (RSBI) is the most commonly used index to predict weaning, there are many studies in the literature recently comparing it with diaphragm ultrasound due to some limitations in its use ( 8 , 9 ). Herein, we aimed to perform diaphragm ultrasonography beside RSBI for predicting the weaning process and its relation with frailty in the critically ill elderly population.

## MATERIALS and METHODS

This study was conducted at the ICU of Ankara Bilkent City Hospital. The study was approved by the Ethical Clearance Committee (Date: 09/10/2022, Number: E2-22-2363). Informed consent was not necessary due to the nature of the study’s retrospective design.

We enrolled consecutive adult patients aged >65 years who were admitted to the ICU between May 2022 and August 2022. Inclusion criteria were as follows: 1 age ≥65 years, 2 respiratory failure requiring mechanical ventilation for more than 48 h. The patients who had intubation history within the last six months were excluded. At ICU admission, these clinical data were collected: age, sex, comorbidities, body mass index (BMI), clinical frailty scale (CFS), reason of ICU admission, acute physiology and chronic health evaluation (APACHE)-II in 24 hour of admission and first day sequential organ failure assessment (SOFA) scores, clinical condition occurred during ICU stay (sepsis, acute respiratory distress syndrome, renal replacement therapy), length of ICU stay, mortality data. We screened malnutrition using geriatric nutritional risk index (GNRI) and modified nutrition risk in critically ill (mNUTRIC) score. Spontaneous breathing trial (SBT) was done by clinician’s decision. Rapid shallow breathing index (RSBI) which is the ratio of respiratory rate to tidal volume was recorded before extubation. Patients were extubated if SBT was tolerated (respiratory rate <35 breaths/min, heart rate<140 beats/min, oxygen saturation ≥90%, 80 mmHg< systolic blood pressure <180 mmHg or <20% change from baseline and absence of increased breathing work or distress signs). Weaning failure was explained as reintubation, mortality within 48 hours after extubation. According to these criteria, patients were put into success or failure groups.

Ultrasound examination was performed immediately at the beginning of SBT, and within the first 48 hours of intubation. High frequency linear probe (Mindray, frequency= 10.7 MHz) was placed between anterior and mid-axillary lines at level of 9-10th intercostal space for Tdi, thickening fraction was calculated by a formula (End-inspiratory Tdi - end-expiratory Tdi/ end-expiratory Tdi). Diaphragmatic excursion (DE) was evaluated during the first SBT. The phased array probe (frequency= 3.1 MHz, gain= 55 dB) was positioned in the anterior subcostal area in the midclavicular line and directed cranially and dorsally by using liver window for DE. Displacement was visualized with M-mode after finding the best view with B-mode. The average value of three consecutive measurements was recorded. After freezing the US image, quantitative parameters were recorded.

## Statistics

The statistical analysis was performed using the statistical software package SPSS 23.0.0.2. Median [interquartile range (IQR)] was used for non-normally distributed data and percentage for categorical variables. The patients were classified into two groups of weaning success and weaning failure. Continuous variables were compared using MannWhitney U-test, and Fisher’s exact test and Chisquared test for categorical comparisons. Spearman test was used for correlation analysis in DE, Tdi and CFS, mNUTRIC, GNRI. Statistical significance was set at 2-sided p< 0.05 for all analyses.

## RESULTS

Thirty-two patients were consecutively enrolled into the study; 18 (56.3%) were females, and mean age (standard deviation) was 79.3 ± 7.9 years. Clinical features are presented in Table 1 . Hypertension, diabetes, and congestive heart failure were the most common comorbidities. Most common reason for ICU admission was coma which was followed by acute respiratory failure.

Eighteen (56.3%) of them was in the weaning failure group. Clinical features were similar in success and failure groups, other than CFS, presence of sepsis and use of vasopressor during ICU stay and mortality ( Table 2 ). According to ultrasound measurements in Table 3 , diaphragmatic excursion before spontaneous breathing trial was the only statistically significant parameter associated with weaning failure [2.37 cm (0.67) vs 1.43 cm (0.15), p= 0.035]. Spearman correlation analysis between CFS, mNUTRIC, GNRI and ultrasound results revealed that baseline Tdi, Tde and DE at SBT were moderately correlated with CFS ( Table 4 ).

**Table 1:** Clinical features of the patients (n= 32)

Mean age (SD)	79.3 (7.9)	BMI, kg/m2, mean (SD)	26.1 (5.4)
Female sex, n (%)	18 (56.3)	Reason of ICU admission, n (%)	
Comorbidities, n (%)		Coma	20 (62.5)
Hypertension	22 (68.8)	Acute respiratory failure	18 (56.3)
Diabetes	11 (34.4)	Sepsis	9 (28.1)
Congestive heart failure	11 (34.4)	APACHE-II, median (IQR)	22.5 (16.2-29.7)
Coronary artery disease	10 (31.3)	SOFA, median (IQR)	7 (5-10.75)
Chronic obstructive lung disease	10 (31.3)	Length of ICU stay, median (IQR)	22.5 (10-36)
Solid organ malignancy	7 (21.9)		

n: Number, SD: Standard deviation, IQR: Interquartile range, BMI: Body mass index, ICU: Intensive care unit, PaO2: Partial pressure of arterial oxygen, FiO2: Fractional inspired oxygen, APACHE: The acute physiology and chronic health evaluation, SOFA: The sequential organ failure assessment.

**Table 2:** Comparison of weaning success and failure groups

	Weaning success (n= 14)	Weaning failure (n= 18)	p value
Age, mean ± SD	80.8 ± 8.1	78.2 ± 7.8	0.36
Female sex, n (%)	7 (50)	11 (61.1)	0.72
BMI, mean ± SD	25.7 ± 3.7	26.5 ± 6.4	0.67
CFS, mean ± SD	6.5 ± 1.7	7.6 ± 0.9	0.035
mNUTRIC, mean ± SD	6.1 ± 2.4	7.2 ± 2.3	0.23
GNRI, mean ± SD	90.5 ± 14.9	93.7 ± 15.8	0.57
APACHE-II, median (IQR)	19 (13.7-30.5)	24.5 (17.7-29.2)	0.30
SOFA, median (IQR)	6.5 (3-8.2)	7.5 (5.7-12)	0.12
Sepsis, n (%)	7 (50)	18 (100)	<0.001
Vasopressor use, n (%)	6 (42.9)	17 (94.4)	0.004
ARDS, n (%)	0	1 (5.6)	>0.99
RRT, n (%)	3 (21.4)	8 (44.4)	0.27
In hospital mortality, n (%)	5 (35.7)	18 (100)	<0.001

n: Number, SD: Standard deviation, BMI: Body mass index, CFS: Clinical frailty scale, mNUTRIC: Modified nutrition risk in critically ill, GNRI: Geriatric nutritional, APACHE: The acute physiology and chronic health evaluation, SOFA: The sequential organ failure assessment, ARDS: Acute respiratory distress syndrome, RRT: Renal replacement therapy.

**Table 3:** Comparison of weaning success and failure groups according to ultrasound measurements

	Weaning success (n= 14)	Weaning failure (n= 18)	p value
RSBI, mean (SD)	70.5 (46)	127.5 (80)	0.09
Baseline DE, cm, mean (SD)	1.71 (0.68)	1.47 (0.62)	0.37
Baseline Tdi, mm, mean (SD)	1.81 (0.30)	1.88 (0.36)	0.58
Baseline Tde, mm, mean (SD)	2.34 (0.40)	2.61 (0.54)	0.18
DE at SBT, cm, mean (SD)	2.37 (0.67)	1.43 (0.15)	0.035
Tdi at SBT, mm, mean (SD)	1.87 (0.25)	1.93 (0.45)	0.76
Tde at SBT, mm, mean (SD)	2.57 (0.39)	2.33 (0.40)	0.36

n: Number, RSBI: Rapid shallow breathing index, SD: Standard deviation, DE: Diaphragm excursion, Tdi: Thickness of diaphragm at end-inspiration, Tde: Thickness of diaphragm at end-inspiration, SBT: Spontaneous breathing trial.

**Table 4:** Correlation between clinical parameters and ultrasound measurements

	CFS	GNRI	mNUTRIC
Baseline DE	Rho=-0.35 p=0.07	-0.10 0.59	0.01 0.94
Baseline Tdi	-0.48 0.013	0.33 0.09	0.047 0.82
Baseline Tde	-0.47 0.015	0.21 0.28	-0.019 0.92
DE at SBT	-0.63 0.011	0.36 0.18	-0.29 0.28
Tdi at SBT	0.018 0.95	0.16 0.55	-0.22 0.42
Tde at SBT	-0.14 0.62	0.04 0.87	-0.35 0.19

CFS: Clinical frailty scale, GNRI: Geriatric nutritional risk index, mNUTRIC: Modified nutrition risk in critically ill, DE: Diaphragm excursion, Tdi: Thickness of diaphragm at end-inspiration, Tde: Thickness of diaphragm at end-inspiration, SBT: Spontaneous breathing trial.

## DISCUSSION

Herein, we showed that, in critically elderly patients, DE before SBT was associated with weaning failure, and it was also associated with patients’ baseline frailty. Baseline thickness of the diaphragm and RSBI before SBT were not associated with weaning failure.

It has previously been demonstrated that sarcopenia impairs the respiratory muscles, and in a systematic review including twenty studies, during weaning from mechanical ventilation and spontaneous breathing trials, both diaphragmatic excursion and diaphragmatic thickening measurements have been used to predict extubation success or failure in general ICU population ( 7 ).

The most often used index to predict weaning is RSBI, which is calculated by dividing respiratory rate by tidal volume ( 10 ). RSBI assesses the balance between the mechanical strain on inspiratory muscles and the muscles’ capacity to adapt to this load during a weaning attempt. Weaning evaluation can still be inaccurate because of the low specificity and positive predictive value of the test ( 11 ). According to the study of Li et al., median diaphragmatic excursion and diaphragm thickening fraction values have been found better in successful weaning group ( 12 ). Diaphragmatic thickening fraction predicted extubation failure better than RSBI. In our study group, RSBI was not statistically different between the weaning groups while excursion values were lower in failure group. That result was consistent with the previous studies revealing better predictive values with diaphragm ultrasound than RSBI ( 8 , 12 ).

Frailty is linked to multiple adverse outcomes, including increased hospital mortality and long-term death, among critically ill patients ( 13 ). Frailty is mainly classified as a coexisting condition with sarcopenia and its association with lower limb muscle mass has been shown in the general ICU and elderly populations before ( 14 , 15 ). Meanwhile, the impact of frailty on respiratory functions in terms of maximal inspiratory and expiratory functions was evaluated ( 16 ). However, the studies investigating diaphragm dysfunction and frailty are limited in the literature. In this study, we showed that baseline thickness of diaphragm and excursion during SBT were associated with baseline frailty.

This study was performed in a single center and in a severe patient population which could limit its generalization to the general ICU population. However, it is important in terms of the information it gives about the use of bedside ultrasound in the elderly group for the prediction of prolonged weaning process which is associated with poor outcome.

In conclusion, ultrasound can be used to show diaphragm dysfunction in the elderly frail population, and a multifactorial approach to the extubation process may include ultrasound instead of using traditional RSBI alone.

## Acknowledgment and/or disclaimers, if any

This research did not receive any specific grant from funding agencies in the public, commercial, or notfor-profit sectors.

## Ethical Committee Approval

This study was approved by the Ankara City Hospital Clinical Research Ethics Committee (Decision no: E2-22-2363, Date: 07.09.2022).

## CONFLICT of INTEREST

The authors declare that they have no conflict of interest.

## AUTHORSHIP CONTRIBUTIONS

Concept/Design: All of authors

Analysis/Interpretation: BE, BM, AA, SB

Data acqusition: BE, BM, AA, SB

Writing: BE

Clinical Revision: BE, CD, DK, ST

Final Approval: All of authors
